# Automated Detection, Segmentation, and Classification of Pleural Effusion From Computed Tomography Scans Using Machine Learning

**DOI:** 10.1097/RLI.0000000000000869

**Published:** 2022-04-02

**Authors:** Raphael Sexauer, Shan Yang, Thomas Weikert, Julien Poletti, Jens Bremerich, Jan Adam Roth, Alexander Walter Sauter, Constantin Anastasopoulos

**Affiliations:** From the Divisions of ∗ Research and Analytical Services; † Cardiothoracic Imaging, Department of Radiology; ‡ Basel Institute for Clinical Epidemiology and Biostatistics, University Hospital Basel, Basel, Switzerland.

**Keywords:** computed tomography, machine learning, pleural effusion

## Abstract

This study trained and evaluated algorithms to detect, segment, and classify simple and complex pleural effusions on computed tomography (CT) scans.

**Materials and Methods:**

For detection and segmentation, we randomly selected 160 chest CT scans out of all consecutive patients (January 2016–January 2021, n = 2659) with reported pleural effusion. Effusions were manually segmented and a negative cohort of chest CTs from 160 patients without effusions was added. A deep convolutional neural network (nnU-Net) was trained and cross-validated (n = 224; 70%) for segmentation and tested on a separate subset (n = 96; 30%) with the same distribution of reported pleural complexity features as in the training cohort (eg, hyperdense fluid, gas, pleural thickening and loculation). On a separate consecutive cohort with a high prevalence of pleural complexity features (n = 335), a random forest model was implemented for classification of segmented effusions with Hounsfield unit thresholds, density distribution, and radiomics-based features as input. As performance measures, sensitivity, specificity, and area under the curves (AUCs) for detection/classifier evaluation (per-case level) and Dice coefficient and volume analysis for the segmentation task were used.

**Results:**

Sensitivity and specificity for detection of effusion were excellent at 0.99 and 0.98, respectively (n = 96; AUC, 0.996, test data). Segmentation was robust (median Dice, 0.89; median absolute volume difference, 13 mL), irrespective of size, complexity, or contrast phase. The sensitivity, specificity, and AUC for classification in simple versus complex effusions were 0.67, 0.75, and 0.77, respectively.

**Conclusion:**

Using a dataset with different degrees of complexity, a robust model was developed for the detection, segmentation, and classification of effusion subtypes. The algorithms are openly available at https://github.com/usb-radiology/pleuraleffusion.git.

Computer-aided quantification and diagnosis systems have become widely available in thoracic radiology, and various pathologies can be automatically detected, segmented and classified on chest radiographs and computed tomography (CT).^1–3^ For pleural disease, effusions can be detected accurately from radiographs, also with deep learning–based image analysis.^4^ However, the occurrence^5^ and amount^6,7^ of effusions are independent prognostic indicators. This became evident in the COVID-19 pandemic when infected patients with pleural effusions had a higher incidence of severe courses, prolonged hospital stays, and higher mortality rates.^8^

Compared with radiography, CT provides accurate pleural effusion quantification; nevertheless, in radiology reports, effusions are commonly described only qualitatively because manual delineation is time-consuming. Automated quantification methods based on traditional image processing or atlas segmentation have resulted in moderate performance, have not included effusion-free control cohorts, or had limited sample sizes.^9,10^

Computed tomography is especially relevant for a detailed assessment of effusion subtypes (ie, hemothorax, empyema, malignant effusion, and pneumothorax) and for detection of the causative diagnosis.^11^ Additional pleural complexity features, such as hyperdense fluid, pleural thickening, gas, and loculation, are used to differentiate between serous and these more complex effusion subtypes^12–14^ (from now on referred to as simple and complex effusions, respectively). This differentiation has implications for patient management^15–17^ and outcome,^18,19^ whereas machine learning models could also be used for CT-guided planning and fast detection of associated periprocedural pneumothorax and hemothorax.^20,21^

## Hypothesis and Purpose

We aimed to develop machine learning models that (1) accurately detect and (2) robustly segment pleural effusions. Our third aim was classification (3) into simple versus complex pleural effusions with random-forest classifiers.

## MATERIALS AND METHODS

The local ethics committee approved this retrospective study (Project ID 2021-00946).

### Study Population

**FIGURE 1:**
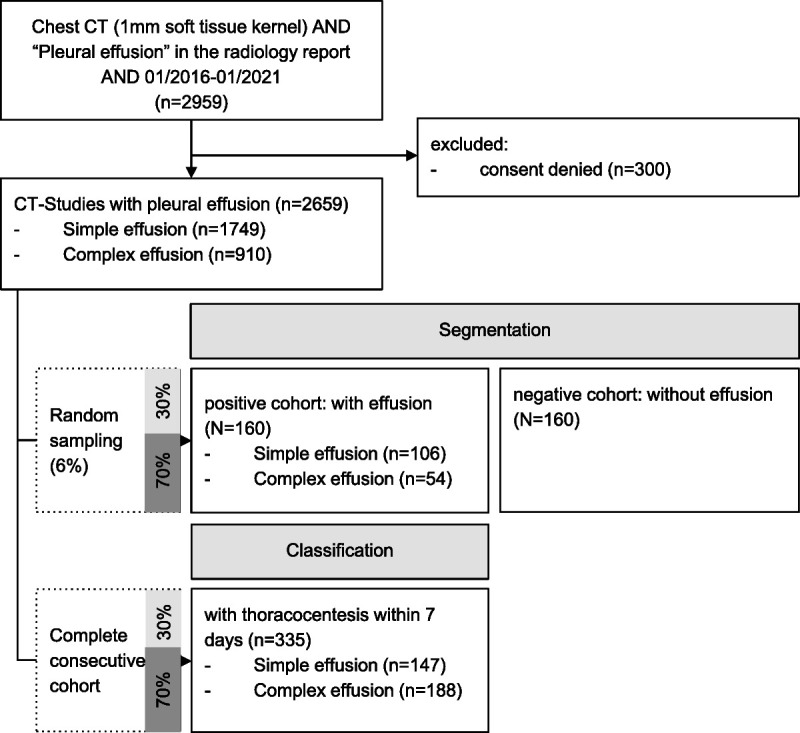
Study flow diagram showing the steps conducted for selection of samples. Complexity features include “hyperdense fluid,” “pleural thickening,” “gas,” and “loculation.” The external Plethora dataset is not included in the flowchart.

The study population consisted of cases with and without pleural effusion, defined as positive and negative cohort, respectively. For the positive cohort, 2659 consecutive patients were retrospectively identified with chest CT scans performed at our tertiary hospital between January 2016 and January 2021 containing the term “pleural effusion” in the radiological report (Fig. 1). We then randomly selected 160 CTs for segmentation of lungs and pleural cavity, preserving the distribution of pleural complexity features as in the whole cohort (Text, Supplementary Digital Content 1, http://links.lww.com/RLI/A689). For the negative cohort, we selected an equal amount of CT datasets (n = 160) from our institutional database as previously described^22^ and conducted a secondary image review for the presence of pleural effusion (reader 1, R.S., postgraduate year [PGY] 4). Our considerations on sample size estimation are provided in Supplementary Digital Content 2 (text), http://links.lww.com/RLI/A690.

After review by reader 1, detection performance was externally validated on all patients in the public National Lung Screening Trial (NLST) dataset^23^ (n = 1061, 2234 CTs with soft tissue kernel). External validation of the segmentation performance was performed using data from the publicly available PleThora project (n = 34),^24^ consisting of manual reference segmentations of the thoracic cavity and effusions in Non-Small-Cell Lung Cancer patients.

For classification of pleural disease, we selected all patients of the positive cohort with biopsy or thoracocentesis within 7 days of the chest CT examination (n = 335).

### CT Acquisition Parameters

Scans were acquired using 3 different CT scanners: Somatom Definition Flash (n = 284, 2 × 128 slice system), Somatom Definition AS+ (n = 262, 128-slice system), and Somatom Definition Edge (n = 109, 128-slice system; all scanners: Siemens Healthineers, Erlangen, Germany). The peak kilovoltage was 120 kVp and an automated tube current modulation was performed. The contrast agent Iopromide (Ultravist 370, Bayer Pharmaceuticals, Berlin, Germany) was administered in 301 (arterial phase, n = 70; biphasic, n = 93; venous, n = 15; CT pulmonary angiography, n = 123; n = 208 in the classification cohort) of the 655 CT studies at a standard injection rate of ~4.0 mL/s and a body weight-adapted volume of up to 120 mL. A soft tissue kernel (30f) of 1.0 mm served as the only input for the algorithm.

### Pleural Effusion Detection and Segmentation

Reader 1 manually segmented the pleural cavity, after processing the original 3-dimensional (3D) chest CTs in a medical image software as previously described.^22^ The segmentations were then exported with separate labels for lung, pleura, and background.

We then divided the segmented CTs (n = 320) into a training/validation (70%, n = 224) and testing dataset (30%, n = 96). During this otherwise random process, we preserved the distribution of complexity feature counts (66.2%, 16.9%, and 16.9% with no, 1, and multiple pleural complexity features, respectively) and the ratio of negative to positive cases (1:1).

To measure interrater variability, 12 studies were randomly selected from the test dataset and were segmented by reader 2 (T.W., in-training, PGY5) and reader 3 (Julien Poletti, in-training, PGY1), who were blinded to the radiology reports. To measure intrarater variability, the same cases were segmented again by reader 1, blinded to and 4 weeks apart from the initial segmentation.

The deep learning model was trained with nnU-Net, which is self-configuring in terms of preprocessing, architecture selection, training, and postprocessing (Table, Supplementary Digital Content 3, http://links.lww.com/RLI/A691).^2^ An ensemble from the 5-fold cross-validation models was used for inference. All processing was performed in Matlab R2018b and Python 3.7.

### Pleural Effusion Classification

We defined effusions with additional pleural complexity features as complex and effusions without complexity features as simple. In the 335 cases for effusion classification, the additional pleural complexity features “hyperdense fluid,” “pleural thickening,” “gas,” and “loculation” were visually determined by reader 1 in consensus with reader 4 (J.B., 29 PGY) and radiologically defined as:

• Hyperdense fluid: Density values greater than 15.6 HU in the pleural cavity,^25^ not otherwise explained, for example, by artifacts. Additional potential indicators such as rib fractures, postoperative changes, pleural fluid sedimentation, or pleural contrast extravasation confirmed the diagnosis, if present.
• Pleural thickening: Nodular or smooth pleural line as seen in the soft tissue kernel.
• Gas: Density values less than −850 within the pleural cavity resembling microbubbles (gas surrounded by pleural fluid) and/or pneumothorax.
• Loculation: Pleural effusion with an obtuse angle to the lung parenchyma (90 degrees < *α* < 180 degrees).

In addition, the classification dataset was dichotomized based on the resulting diagnosis of serous effusion from biopsy or thoracocentesis in the test dataset. However, microscopic evidence of erythrocytes in an otherwise serous effusion was not rated as a complex effusion, as this can be periprocedural.

The sample was randomly split into training/validation and testing datasets (n = 234 and n = 101, 70% and 30%, respectively).

### Interpretable Complexity and Radiomic Features

**FIGURE 2:**
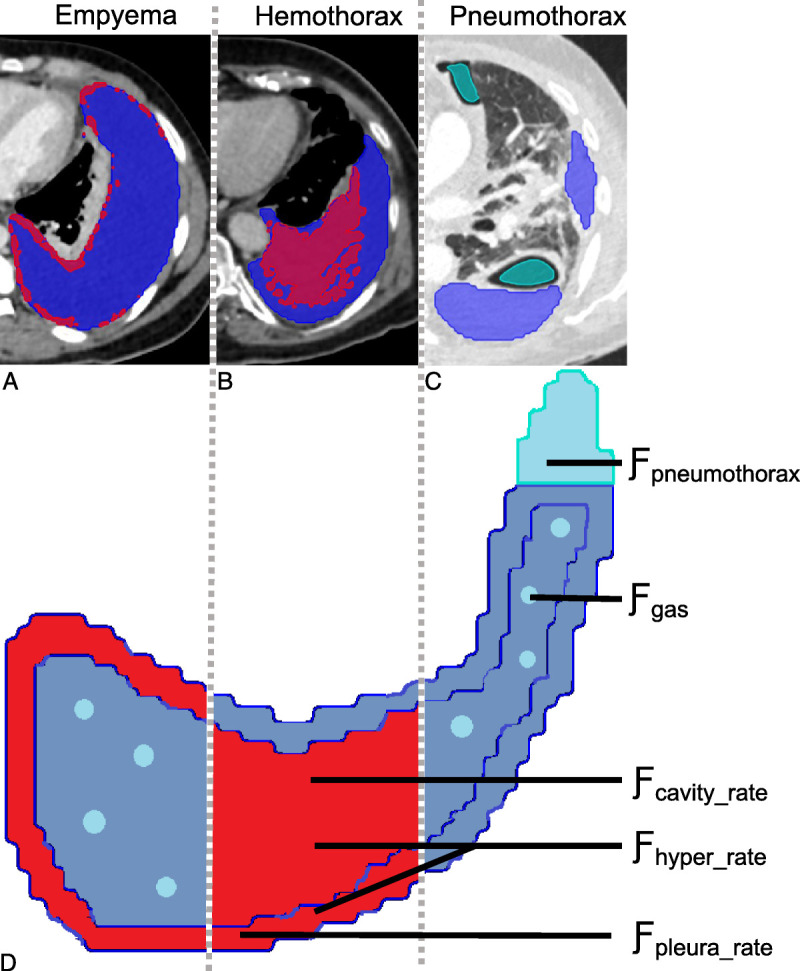
Automated segmentation (dark blue) and applied thresholds (>30 HU and >2 mL in red, <−850 HU in light blue) in exemplary subjects with (A) empyema, (B) hemothorax, and (C) pneumothorax. D, Schematic overview of the derived pleural complexity features. A, The pleural thickening results in a slight hyperdensity of the total pleural segmentation (hyper_rate: 13%), which is concentrated in the pleura (pleura_rate: 43%) and not in the pleural cavity (cavity_rate: 5%), resulting in a low in:out ratio (0.11). B, The blood in the pleural cavity leads to a high total hyperdensity (hyper_rate: 34%), predominantly in the cavity (cavity_rate: 37%), resulting in a higher inout_rate (1.33). C, The patient with pneumothorax had a gas volume of 50 mL and pleural effusion of 686 mL.

Receiver operating characteristic (ROC) analysis was used to define the lower threshold with the highest area under the curve (AUC) for hyperdense fluid (thresholds: 8.5 HU,^26,27^ 15.6 HU,^25^ and 30.0 HU^28^; AUCs: 0.60, 0.62, and 0.63, respectively) and pleural margin thickness (upper threshold: 4 mm,^29^ 5 mm,^30^ and 8 mm^31^; AUCs: 0.59, 0.59, and 0.57, respectively). A minimal volume of 2 mL was set as a prerequisite for all features to exclude spurious hyperdensities. Based on the resulting thresholds of the ROC analysis (30 HU and 4 mm), we defined the following features, summarized in Figure 2 and Supplementary Digital Content 4 (Table), http://links.lww.com/RLI/A692: Ƒ_hyper_ (absolute hyperdense volume), Ƒ_hyper_rate_ (hyperdensity rate in %), Ƒ_pleura_rate_ (hyperdensity rate of the pleura), Ƒ_cavity_rate_ (cavity), their ratio Ƒ_inout_ratio_ = Ƒ_cavity_rate_ / Ƒ_pleura_rate_ and indexed ratio Ƒ_inout_ratio_index_ = Ƒ_inout_ratio_ * Ƒ_hyper_rate_. For gas quantification (upper threshold: −850 HU), we defined 2 features: gas within the pleural segmentation (Ƒ_gas_) and gas in pneumothorax (Ƒ_pneumothorax_), latter as gas adjacent but outside lung and pleural segmentation and without connection to the bronchial system. Furthermore, we used all radiomic features from the Python package PyRadiomics (version 3.0.1).^32^

### Classification Models

Random forest models (for details: Table, Supplementary Digital Content 5, http://links.lww.com/RLI/A693) were used for classification into simple and complex effusion as well as for the prediction of any underlying pleural complexity features (“hyperdense fluid,” “pleural thickening,” “gas,” and “loculation”), resulting in a total of 5 models. Initially, the count of positive and negative cases for each classification task was inevitably unbalanced; therefore, the datasets were randomly downsampled to a 1:1 ratio. For each class, preliminary training was performed to select the most informative variables (50 percentile of feature importance of both interpretable complexity and radiomic features). Then, with the most important half of the variables, the models were further fine-tuned with a leave-one-out cross-validation. Finally, a model was trained for each of the 5 classification tasks and was evaluated on the test data with a test-positivity threshold greater than 0.5.

### Statistical Analysis

To evaluate pleural effusion detection and classification performance, we used sensitivity, specificity, negative predictive value (NPV), positive predictive value, and ROC analysis. We used nonparametric tests to evaluate intergroup differences (Mann-Whitney *U* test for 2 variables and Kruskal-Wallis test for more than 2 variables). To evaluate the performance of the segmentation algorithm, we used Dice coefficient and intraclass correlation coefficient (ICC) to compare with human intrarater and interrater variability. Volumetric results were compared with Bland-Altman analysis and linear correlation with the Pearson coefficient.

For classification, diagnostic accuracy measures are reported separately both for the radiological absence or presence of pleural complexity features (simple and complex effusion, respectively) and based on reports from biopsy or thoracocentesis (serous effusion as simple; presence of pleural empyema or pleural carcinomatosis as complex effusion).

A *P* value of <0.05 was considered statistically significant. All statistical analyses were performed in R 4.0.5 (R Core Team, Vienna, Austria). All results in the main text refer to the respective test datasets for segmentation and classification, whereas the respective results of the cross-validation for detection (Table, Supplementary Digital Content 6, http://links.lww.com/RLI/A694), segmentation (Table, Supplementary Digital Content 7, http://links.lww.com/RLI/A695), and classification (Table, Supplementary Digital Content 8, http://links.lww.com/RLI/A696) are summarized in the supplement.

## RESULTS

### Study Population

The mean age of patients with pleural effusion (n = 2659) was 68.39 years (range, 18–102 years), with 1076 women and 1583 men. Related to all pleural effusions, 66% (n = 1749) had no pleural complexity feature, 17% (n = 446) had 1 pleural complexity feature, and 17% (n = 464) had multiple complexity features, and these ratios were preserved in the segmentation subsets. The mean age in the segmentation dataset (n = 320) was 63.42 years (range, 18–97 years; 136 women) and in the classification dataset (n = 335) was 68.64 years (range, 18–96; 125 women). In both samples, training/validation and test datasets did not significantly differ regarding patients’ age (*P* = 0.360) and effusion volume (*P* = 0.192).

Based on the manual reference standard of the segmentation (n = 160), 74 of the 160 CT examinations showed bilateral pleural effusions. The total effusion volume ranged between 2–2318 mL (mean [SD], 285 [402] mL; median, 131 mL) in the cross-validation and 5–2094 mL (mean [SD], 469 [499] mL; median, 332 mL) in the test dataset. In the test segmentation dataset, 3 had hyperdense fluid, 5 had pleural thickening, 5 had gas, and 5 were loculated. The sample size of the test dataset was confirmed after testing the ensemble of models with an ICC of 0.993 (95% confidence interval [CI], 0.98–1.00; power, 0.90). Therefore, the following accuracy and performance measures are based on the test dataset.

In the classification cohort, 147 patients had simple pleural effusions (no pleural complexity feature) and 188 patients had complex effusions (1 complexity feature, n = 84; multiple complexity features, n = 104), with a total of n = 17 with hyperdense fluid, n = 95 with pleural thickening, n = 100 with gas, and n = 128 with loculation. Of the 208 CT studies with contrast agent administration, 63 patients had visible pleural enhancement.

### Detection of Pleural Effusion

**FIGURE 3:**
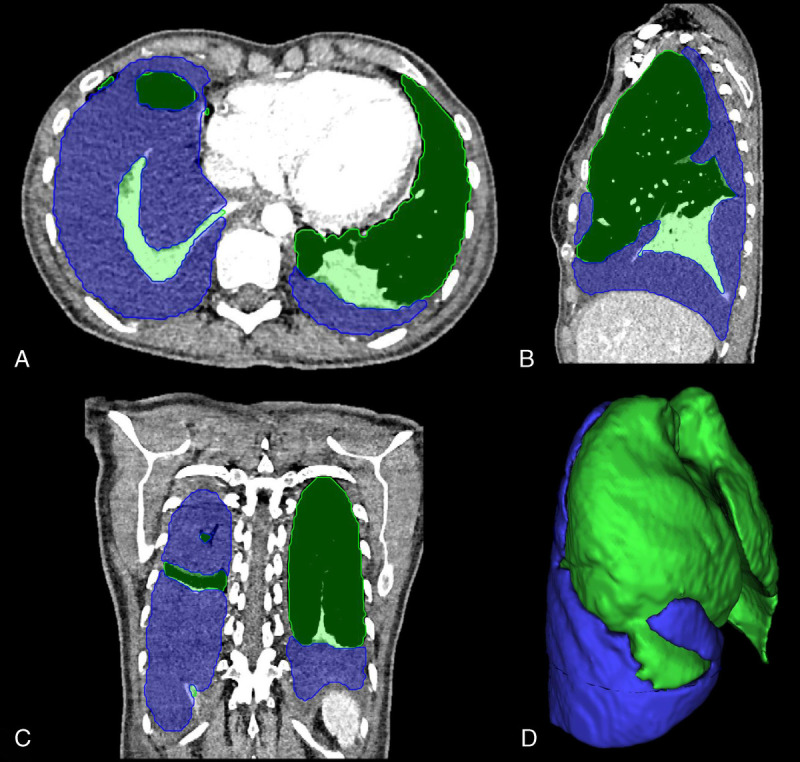
(A) Axial, (B) sagittal, (C) coronal plane, and (D) isosurface of model prediction for pleural effusion and lung of a 54-year-old female patient with an empyema on the right side and a pleural effusion on the left side. Dark green is well-aerated lung parenchyma, whereas light green is atelectasis.

With the radiological reports as the reference standard, the sensitivity for detection of pleural effusion was 0.99 (95% CI, 0.91–1.00) and the specificity was 0.98 (95% CI, 0.95–1.00). The AUC for the segmentation cohort (both validation and test data) was 0.996 (95% CI, 0.97–1.00). Figure 3 shows an example of segmentation and Table 1 summarizes the diagnostic accuracy measures. Failure analysis of incorrectly classified cases can be found in Supplementary Digital Content 9 (Figure), http://links.lww.com/RLI/A697.

**TABLE 1:** Diagnostic Accuracy Measures for Detection of Pleural Effusion

	Per Patient	Per Pleural Effusion
True-positive	48	72
False-negative	0	1
True-negative	47	118
False-positive	1	1
Sensitivity (95% CI)	1.00 (0.91–1.00)	0.99 (0.91–1.00)
Specificity (95% CI)	0.98 (0.88–1.00)	0.99 (0.95–1.00)
Positive predictive value (95% CI)	0.98 (0.88–1.00)	0.99 (0.92–0.99)
Negative predictive value (95% CI)	1.00 (0.91–1.00)	0.99 (0.95–0.99)

CI indicates confidence interval.

On the external NLST dataset (2234 eligible CTs), the model performed with a sensitivity of 1.00 (95% CI, 0.94–1.00) and a specificity of 0.99 (95% CI, 0.989–0.997).

### Segmentation of Pleural Effusion

Inference on the test dataset showed a mean (SD) Dice coefficient of 0.84 (0.16) (95% CI, 0.80–0.88; median, 0.89). The mean (SD) absolute volume difference was 33 (53) mL (95% CI, 20–45 mL; median, 13 mL), with a significant linear correlation between manual segmentation and predicted volume (Pearson *r* = 0.996, *P* < 0.001).

On the external PleThora (n = 34; mean volume, 383 mL; 95% CI, 278–487 mL) dataset, the model performed with a median Dice coefficient of 0.71 with an intraclass correlation of 0.97 (95% CI, 0.95–0.99; *P* < 0.001) for pleural effusion volume and a mean (SD) absolute volume difference of 87 (67) mL (95% CI, 63–110 mL; median, 90 mL). Lung segmentation performance (Dice: mean, 0.97; 95% CI, 0.96–0.99; median, 0.99) did not significantly differ between cases with and without effusion (*z* score: −0.70, *P* = 0.480).

### Intrarater and Interrater Agreement

There was an excellent ICC between the human readers (0.97; 95% CI, 0.90–0.99), with a higher ICC between the manual reference standard and the automated segmentation (1.00; 95% CI, 0.99–1.00), which is comparable to the intrarater agreement (1.00; 95% CI, 0.91–1.00). Dice coefficients of intrarater segmentation (0.85; 95% CI, 0.81–0.89) and the automated segmentation (0.84; 95% CI, 0.79–0.89) were comparable (*r* = 0.96, *P* < 0.001, n = 12). Supplementary Digital Content 10 (Figure), http://links.lww.com/RLI/A698, summarizes the intrarater and interrater agreement between the automated segmentation compared with the reference standard.

### Probable Confounding Factors for the Performance of Segmentation

Although the dice coefficient increased with the volume of the effusion (linear regression, analysis of variance: *F* = 35.60; test: *F* = 15.62; *P* < 0.001), no other factor could be identified that influenced the model’s performance. In the test dataset, neither the presence of additional pleural complexity features (Kruskal-Wallis: 0.36, *P* = 0.837) nor the previous application of a contrast agent (*z*: −1,9, *P* = 0.060) showed any influence on the segmentation. Supplementary Digital Content 11 (Figure), http://links.lww.com/RLI/A699, visually summarizes the results for the probable confounding factors.

### Classification of Pleural Effusion

**FIGURE 4:**
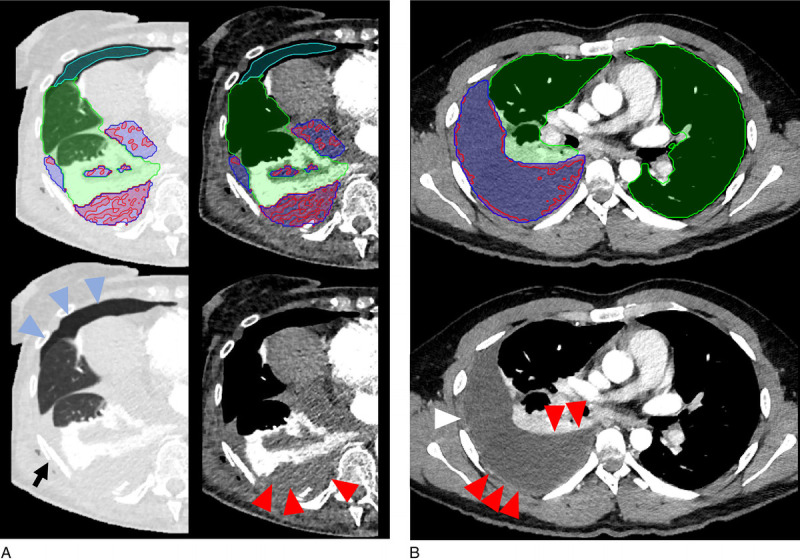
Classification results in 2 selected cases. Model predictions are shown at the top and original images at the bottom row. A, Posttraumatic chest CT of a patient with several rib fractures (black arrow), pneumothorax (blue arrowheads/light blue mask), and a complex effusion on the right (blue mask) with hyperdense fluid (red arrowheads and red mask) and with atelectasis of the lower lobe (green mask). B, Chest CT of a patient with empyema on the right side (blue) and no effusion on the left side. The hyperdense outer pleural space (HU >30 threshold, red mask) emphasizes the pleural thickening (red arrowheads).

The initial training of the classification models identified all interpretable complexity features as informative features. Figure 4 shows 2 examples of model input. From the radiomics features, mostly pleural “shape features” (elongation, flatness, least axis length, maximum 2D diameter, maximum 2D diameter, mesh volume, minor axis length, sphericity, surface area, and surface volume ratio and voxel volume) were integrated during the preliminary training. The most informative features depended on the classification task and were Ƒ_pleura_rate_ and Ƒ_hyper_rate_ for “pleural thickening”; Ƒ_inout_rate_, Ƒ_inout_ratio_index_ for “hyperdense fluid”; *Neighborhood Grey Tone Difference Matrix (NGTDM) strength* and *NGTDM busyness* for “gas”; and Ƒ_hyper_rate_ and Ƒ_inout_ratio_index_ for “loculation” (see Table, Supplementary Digital Content 12, http://links.lww.com/RLI/A700).

**FIGURE 5:**
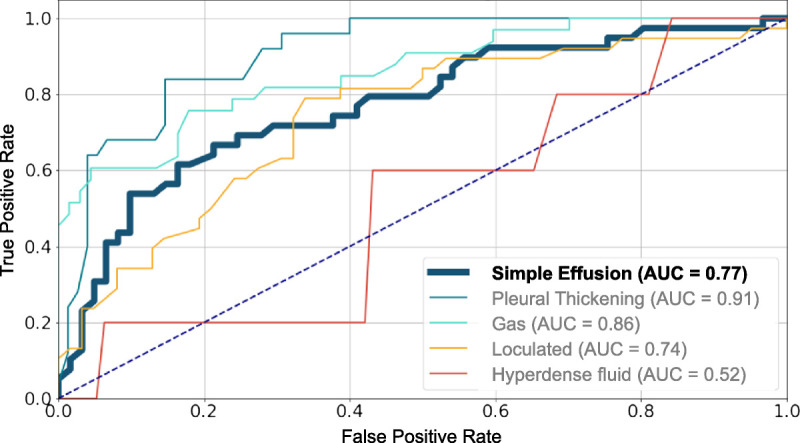
ROC related to the entire test dataset classification cohort (n = 101). The thick blue line represents the prediction for “simple effusion.” For cases with additional pleural complexity features, the respective ROCs of the 4 models are shown. The number of positive features in the test dataset is 39 for “no complexity feature,” 25 for “pleural thickening,” 33 for “gas,” 38 for “loculation,” and 5 for “hyperdense fluid.”

Compared with the radiological reference standard, diagnostic accuracy for “simple effusion” had a sensitivity of 0.67 (95% CI, 0.51–0.79), a specificity of 0.75 (95% CI, 63.3–84.5), an NPV of 0.78 (95% CI, 0.65–0.87), and an AUC of 0.77. Regarding the classification tasks for the 4 pleural complexity features (hyperdense fluid, pleural thickening, gas, and loculation) used for the distinction into simple and complex effusion, results are summarized in Table 2, with relatively high NPVs ranging from 0.78 (loculation) to 0.94 (hyperdense fluid and pleural thickening) and with an AUC ranging from 0.52 (hyperdense fluid) to 0.91 (pleural thickening) in the corresponding ROC curves (see Fig. 5; for ROC curves of cross-validation, see Supplementary Digital Content 13, http://links.lww.com/RLI/A701).

**TABLE 2:** Diagnostic Accuracy Measures for Classification of Pleural Effusion

	Pleural Complexity Features				Simple Effusion
	Hyperdense Fluid	Pleural Thickening	Gas	Loculation	
True-positive	1	21	25	26	26
False-negative	4	4	8	12	13
True-negative	58	62	52	42	46
False-positive	37	13	15	20	15
Sensitivity (95% CI)	0.20 (0.04–0.62)	0.84 (0.65–0.94)	0.76 (0.59–0.87)	0.68 (0.53–0.81)	0.67 (0.51–0.79)
Specificity (95% CI)	0.61 (0.51–0.70)	0.83 (0.73–0.90)	0.78 (0.66–0.86)	0.68 (0.55–0.78)	0.75 (0.63–0.85)
Positive predictive value (95% CI)	0.03 (0.00–0.15)	0.62 (0.44–0.77)	0.63 (0.46–0.77)	0.57 (0.41–0.71)	0.63 (0.47–0.77)
Negative predictive value (95% CI)	0.94 (0.84–0.98)	0.94 (0.84–0.98)	0.87 (0.75–0.94)	0.78 (0.64–0.88)	0.78 (0.65–0.87)

CI indicates confidence interval.

Compared with reports from biopsy or thoracocentesis, the model detected simple effusions with a sensitivity of 0.97 (95% CI, 0.86–1.00) and a specificity of 0.64 (95% CI, 0.51–0.76).

## DISCUSSION

We developed and comprehensively analyzed an algorithm for the automated detection and segmentation of pleural effusions and introduced strategies for the classification between simple and complex pleural effusions. A highly sensitive detection (0.99; 95% CI, 0.91–1.00) and a robust segmentation (Dice: 0.84; 95% CI, 0.80–0.88) were achieved. The classification between simple and complex pleural effusion resulted in a modest sensitivity of 0.67 and a moderate specificity of 0.75, whereas the random-forest algorithms incorporated both radiomics and radiologically interpretable complexity features, such as density values and their distribution in the pleural cavity.

First, the performance of a widely adopted deep learning-based segmentation method^2^ was tested in a clinical dataset, systematically containing both simple and complex pleural effusions, as well as patients without effusions. An accurate detection rate was also shown in the external NLST dataset.^23^ Similar to other deep learning–based nonpleural segmentation tasks,^33–35^ the detection and segmentation accuracy was high, irrespective of effusion complexity, laterality, effusion volume, and previous application of contrast agents. Previously, computer vision methods have been used for automated pleural effusion segmentation on limited CT sample sizes.^9,10^ The proposed segmentation algorithm provides robust volumetric results in a large and heterogeneous clinical sample and therefore might have implications for clinical use and offers the potential for prognostication.^19^ The segmentation algorithm was validated on the PleThora dataset,^24^ consisting of tumor-associated effusions, and provided a good volumetry with an ICC of 0.97. Dice coefficient and absolute volume difference were inferior compared with the test dataset, partially explained by inconsistencies of human-delineated segmentations in the PleThora dataset, whereas our algorithm tends to primary segment similar densities. Previously, effusions have been detected and (semiquantitatively) quantified in chest radiography,^36^ although sonography is superior in detecting effusions, which in turn is limited in effusion volumetry compared to CT.^37,38^ In contrast, if applied broadly and systematically, our proposed algorithm has the potential to be utilized for reliable follow-up measurements.

Second, for the classification of pleural effusions, we defined “complex” effusions as opposed to serous or “simple” effusions. The former category subsumes various pleural diagnoses (ie, hemothorax, empyema, malignant effusion, and pneumothorax), which radiologically have partially overlapping, complexity features,^39,40^ often used in decision making^15–17^ and prognostication.^18,40^ The classification task identified the prespecified complexity features as informative, whereas the addition of the radiomic features further leveraged diagnostic accuracy. The classification between simple and complex effusions showed a moderate performance with an AUC of 0.77, whereas classification for the separate pleural features ranged between an AUC of 0.52 for hyperdense fluid and an AUC of 0.91 for pleural thickening. This can be partially explained by the moderate diagnostic accuracy of the CT with its predominantly high specificity and lower sensitivity for different pleural diseases.^12,41^ The relatively high NPVs can aid in the identification of complex pleural effusions, yet the low positive predictive values indicate the necessity of a radiological evaluation. Still, an objectifying visualization of the automated results is pivotal to familiarize radiologists with automated (yet non-black-box) tools, as we have previously shown in other volumetric tasks.^42^

The introduction of shape and textural features has been proposed to overcome the varying interrater agreements with regard to the classification of complex pleural lesions.^43^ Interestingly, in the present study, most of the radiomic features were discarded in the pretraining selection step, whereas the predefined, interpretable complexity features were more relevant for the classification tasks. Similarly, classification of tumor grade prediction has previously achieved higher AUC with prespecified features based on “traditional” radiological characteristics compared with a radiomics-based model, whereas their combination showed the highest diagnostic accuracy.^44^ The preference of our classification models for traditional features of pleural complexity is contributing to the ongoing discussion about the applicability of radiomics in CT.^42,45^ In future research, automated pleural segmentation and classification might also contribute to better prognostication, that is, identification of treatment responders from diaphragm shape analysis.^46^

There are several limitations to our work. First, eligible patients were retrospectively selected on scanners of 1 vendor at a single institution. The models’ performance on examinations acquired with different scanners might differ. However, a similarly small sample size of CT scans from a new site might serve for training a custom segmentation nnU-Net model, after adopting the settings as shown in Supplementary Digital Content 3, http://links.lww.com/RLI/A691. Second, reference standards for segmentation and classification were based mainly on imaging. Nevertheless, the reported features had been validated by at least 3 radiologists (one of which was board certified). Third, the absolute number of patients with hemothorax in the classification cohort was relatively low. This was probably the cause for the low diagnostic accuracy of the classification algorithm for hyperdense fluid, which could be improved in the future by increasing the sample size.

### Implications for Practice

Automatic detection and robust segmentation of pleural effusions in chest CTs allow for routine use without interaction, 3-dimensional volumetry, and rapid quantification. The proposed classification can be used to identify pleural effusions with and without pleural complexity features, and thus, radiologists can be aided in the diagnoses of patients with empyema, hemothorax, or pneumothorax. The trained models are openly available on a public repository.

## Supplementary Material
