# Supplementary material for: Automated Detection, Segmentation, and Classification of Pleural Effusion From Computed Tomography Scans Using Machine Learning
Source: Invest Radiol. 2022 Apr 2;57(8):552–9. doi: 10.1097/RLI.0000000000000869 (PMC9390225; doi:10.1097/RLI.0000000000000869)
Supplement: Supplementary file 1 [file ir-57-552-s001.docx]

**Supplemental Digital Content 4: Feature definition**

| **Feature** | **Definition** |
| --- | --- |
| Ƒ_hyper_ (in ml) | Hyperdense content of the pleural segmentation (> 2 ml and >30 HU) |
| Ƒ_hyper_rate_ | $Ƒ_{hyper}$/(total pleural segmentation volume) |
| Ƒ_pleura_rate_ | $Ƒ_{hyper}$in pleural margin of 4mm/(total pleural segmentation volume) |
| Ƒ_cavity_rate_ | $Ƒ_{hyper}-{(Ƒ}_{hyper}$in pleural margin of 4mm)/(total pleural segmentation volume) |
| Ƒ_inout_ratio_ | Ƒ_cavity_rate_/Ƒ_pleura_rate_ |
| Ƒ_inout_ratio_index_ | Ƒ_inout_ratio_  * Ƒ_hyper_rate_ |
| Ƒ_gas_ (in ml) | Gas content (<-850 HU) in the pleural segmentation (irrespective of volume). |
| Ƒ_pneumothorax_ (in ml) | Gas in direct contact to lung segmentation or pleural segmentation minus gas with connection to the bronchial system |
