# Supplementary material for: Automated Detection, Segmentation, and Classification of Pleural Effusion From Computed Tomography Scans Using Machine Learning
Source: Invest Radiol. 2022 Apr 2;57(8):552–9. doi: 10.1097/RLI.0000000000000869 (PMC9390225; doi:10.1097/RLI.0000000000000869)
Supplement: Supplementary file 2 [file ir-57-552-s002.docx]

**Supplemental Digital Content 5:** **Configuration of the classification model**

| **Design** | **Parameter** |
| --- | --- |
| Number decision trees | 100 |
| Number of features for pretraining | 108 |
| Max number of features considerd for splitting a node | Auto |
| Number features considered | 54 |
| Cross-Validation | leave-one-out |
| Method for sampling data points | bootstrap |
| Variables | Prior defined pleural features |
| Maximum number of levels in each decision tree | None |
| Minimum number of data points placed in a node before the node is split | 2 |
| Minimum number of data points allowed in a leaf node | 1 |
| Maximum depth of the individual trees | None |
