# Supplementary material for: Automated Detection, Segmentation, and Classification of Pleural Effusion From Computed Tomography Scans Using Machine Learning
Source: Invest Radiol. 2022 Apr 2;57(8):552–9. doi: 10.1097/RLI.0000000000000869 (PMC9390225; doi:10.1097/RLI.0000000000000869)
Supplement: Supplementary file 3 [file ir-57-552-s003.docx]

**Supplemental Digital Content 6: Pleural effusion detection based on cross-validation**

| **cross-validation** | **Per patient** | **Per pleural effusion** |
| --- | --- | --- |
| **true positive** | 111 | 162 |
| **false negative** | 1 | 4* |
| **true negative** | 91 | 241 |
| **false positive** | 21 | 41 |
| **sensitivity** | 0.99 (0.94-1.00) | 0.98 (0.94-0.99) |
| **specificity** | 0.81 (0.73-0.88) | 0.85(0.81-0.89) |
| **positive predictive value** | 0.84 (0.76-0.90) | 0.80 (0.73-0.85) |
| **negative predictive value** | 0.99 (0.93-1.00) | 0.98 (0.96-0.99) |
| **n:** | Patients: 112 | Effusions: 166 |

* one unilateral and 3 with bilateral effusion.
