# Supplementary material for: Automated Detection, Segmentation, and Classification of Pleural Effusion From Computed Tomography Scans Using Machine Learning
Source: Invest Radiol. 2022 Apr 2;57(8):552–9. doi: 10.1097/RLI.0000000000000869 (PMC9390225; doi:10.1097/RLI.0000000000000869)
Supplement: Supplementary file 4 [file ir-57-552-s004.docx]

**Supplemental Digital Content 7: Pleural effusion segmentation based on cross-validation**

| **Cross-validation: means (95% CI)** | **Per patient** | **per pleural effusion** |
| --- | --- | --- |
| **Dice coefficient** | 79.0 (95% CI:76.7-81.3) | 77.6 (95% CI: 75.3-79.8) |
| **Absolute volume difference** | 52.0 ml (95% CI:31.6-72.5) | 36.8 ml (95 CI: 22.9-50.7) |
| **n:** | Patients: 112 | Effusions: 166 |
