# Supplementary material for: Automated Detection, Segmentation, and Classification of Pleural Effusion From Computed Tomography Scans Using Machine Learning
Source: Invest Radiol. 2022 Apr 2;57(8):552–9. doi: 10.1097/RLI.0000000000000869 (PMC9390225; doi:10.1097/RLI.0000000000000869)
Supplement: Supplementary file 5 [file ir-57-552-s005.docx]

**Supplemental Digital Content 8: Classification based on cross-validation**

|  |  |  |  |  |  |  |  |  |
| --- | --- | --- | --- | --- | --- | --- | --- | --- |
|  |  |  |  |  |  |  |  |  |
|  |  |  |  |  |  |  |  |  |
|  |  |  |  |  |  |  |  |  |
|  |  |  |  |  |  |  |  |  |
|  |  |  |  |  |  |  |  |  |

|  | **Pleural complexity features** | | | | **Simple effusion** |
| --- | --- | --- | --- | --- | --- |
|  | Blood | Pleural thickening | Gas | Loculation |  |
| **True Positive** | 8 | 44 | 50 | 68 | 83 |
| **False Negative** | 4 | 9 | 17 | 25 | 23 |
| **True Negative** | 127 | 140 | 120 | 112 | 102 |
| **False Positive** | 96 | 42 | 48 | 30 | 27 |
| **Sensitivity (95% CI)** | 0.67 (0.39-0.86) | 0.83 (0.71-0.91) | 0.75 (0.63-0.84) | 0.73 (0.63-0.81) | 0.78 (0.70-0.85) |
| **Specificity (95% CI)** | 0.57 (0.50-0.63) | 0.77 (0.70-0.82) | 0.71 (0.64-0.78) | 0.79 (0.71-0.85) | 0.79 (0.71-0.85) |
| **DOR (95% CI)** | 2.6 (0.0-0.9) | 16.3 (7.4-36.1) | 7.4 (3.9-14.0) | 10.2 (5.5-18.7) | 13.6 (7.3-25.5) |
