# Supplementary material for: Automated Detection, Segmentation, and Classification of Pleural Effusion From Computed Tomography Scans Using Machine Learning
Source: Invest Radiol. 2022 Apr 2;57(8):552–9. doi: 10.1097/RLI.0000000000000869 (PMC9390225; doi:10.1097/RLI.0000000000000869)
Supplement: Supplementary file 6 [file ir-57-552-s006.docx]

## **Supplementary Digital Content 1: Explains grouping of CT reports based on pleural complexity features**

Reader 1 (R.S.; in-training; 4th post-graduate year) extracted the following CT findings from the written findings radiological reports: “hyperdense fluid", "pleural thickening", "gas bubbles or pneumothorax", "loculation", "pleural plaques, calcification or nodularity", "chest tube" and "subdiaphragmatic fluid". Based on the feature count the examinations were divided into three groups: No additional pleural complexity feature(i.e. simple effusion), one additional complexity feature, and >1 complexity feature. Patients with bilateral pleural effusion were assigned to a category based on the more complex side.
