# Supplementary material for: Automated Detection, Segmentation, and Classification of Pleural Effusion From Computed Tomography Scans Using Machine Learning
Source: Invest Radiol. 2022 Apr 2;57(8):552–9. doi: 10.1097/RLI.0000000000000869 (PMC9390225; doi:10.1097/RLI.0000000000000869)
Supplement: Supplementary file 7 [file ir-57-552-s007.docx]

**Supplementary Digital Content 2: Explains Sample Size Estimation for the Test dataset**

After completing the cross-validation of the segmentation, we decided to check the intended test dataset size for the segmentation algorithm based on a power analysis: The inter-class correlation of the volumes between manual segmentation and the predicted segmentation of the model in cross-validation is 0.97 (95% CI: 0.96-0.98). Based on the test dataset size of 72 effusions from the 48 patients, it can be expected that with a power of 90% the ICC of the test dataset is greater than 0.96 with a probability of 95.3 %.
