# Supplementary material for: Automated Detection, Segmentation, and Classification of Pleural Effusion From Computed Tomography Scans Using Machine Learning
Source: Invest Radiol. 2022 Apr 2;57(8):552–9. doi: 10.1097/RLI.0000000000000869 (PMC9390225; doi:10.1097/RLI.0000000000000869)
Supplement: Supplementary file 8 [file ir-57-552-s008.docx]

**Supplemental Digital Content 3: Configuration of the segmentation model**

| **Design** | **Parameters** |
| --- | --- |
| Image preprocessing | Image downsampling to 2x2x2 mm^3^ |
| Hard-/Software | Matlab R2018b and Python 3.7 on a workstation with a consumer-grade graphic processor unit (Nvidia RTX 2080Ti). |
| Optimizer | SGD with Nesterov momentum (µ=0.99) |
| Learning rate | Poly-learning rate schedule (initial 0.01) |
| Data augmentation | Gaussian noise and blur, brightness, contrast, simulation of low resolution, gamma correction and mirroring |
| Loss function | Dice and cross-entropy |
| Training procedure | 1000 epochs x 250 minibatches, foreground oversampling |
| Inference procedure | Sliding window with half-patch size overlap. Gaussian patch center weighting |
| Architecture template | Encoder-decoder with skip connection, instance normalization, leaky ReLU, deep supervision |
| Intensity normalization | Global dataset percentile clipping, z-score with global foreground mean and s.d. |
| Image resampling strategy | In-plane with third-order spline, out- of-plan with nearest neighbor |
| Annotation resampling strategy | Nearest neighbor interpolation to original spatial resolution |
| Image target spacing | Lowest resolution axis tenth percentile |
| Patch size | [128, 128, 128] |
| Batch size | 2 |
| Ensemble selection | 3D U-Net according to cross-validation performance |
