# Supplementary material for: Automated Detection, Segmentation, and Classification of Pleural Effusion From Computed Tomography Scans Using Machine Learning
Source: Invest Radiol. 2022 Apr 2;57(8):552–9. doi: 10.1097/RLI.0000000000000869 (PMC9390225; doi:10.1097/RLI.0000000000000869)
Supplement: Supplementary file 9 [file ir-57-552-s009.docx]

**Supplementary Digital Content 12: Five most informative features for each classification task**

| Importance | blood | Pleural thickening | gas | loculation | simple effusion |
| --- | --- | --- | --- | --- | --- |
| 1 | Ƒ_inout_ratio_  (0.10) | Ƒ_pleura_rate_  (0.12) | original_ngtdm_Strengh  (0.04) | Ƒ_hyper_rate_  (0.06) | Ƒ_hyper_rate_  (0.07) |
| 2 | Ƒ_inout_ratio_index_  (0.10) | Ƒ_hyper_rate_ (0.11) | original_ngtdm_Busyness  (0.04) | Ƒ_inout_ratio_index_  (0.06) | original_firstorder_90Percentile (0.06) |
| 3 | Ƒ_cavity_rate_ (0.07) | originale_firstorder_90Percentile(0.09) | original_glcm_SumSquares  (0.04) | Ƒ_pleura_rate_  (0.05) | Ƒ_inout_radio_index_  (0.05) |
| 4 | original_gldm_LargeDependenceLowGrayLevelEmphysis (0.06) | Ƒ_hyper_ (0.06) | original_shape_SurfaceVolumeRatio (0.04) | original_firstorder_Median (0.04) | Ƒ_pleura_rate_  (0.05) |
| 5 | original_gldm_SmallDependenceHighGrayLevelemphysis (0.05) | Ƒ_cavity_rate_  (0.05) | original_firstorder_Minimum (0.03) | Ƒ_cavity_rate_ (0.04) | Ƒ_cavity_rate_  (0.05) |
| ... | ... | ... | ... | ... | ... |

The table summarizes the five most informative features for the prediction of each pleural finding. Mean decrease in impurity in brackets.
