# Supplementary figures and images for: Automated Detection, Segmentation, and Classification of Pleural Effusion From Computed Tomography Scans Using Machine Learning
Source: Invest Radiol. 2022 Apr 2;57(8):552–9. doi: 10.1097/RLI.0000000000000869 (PMC9390225; doi:10.1097/RLI.0000000000000869)

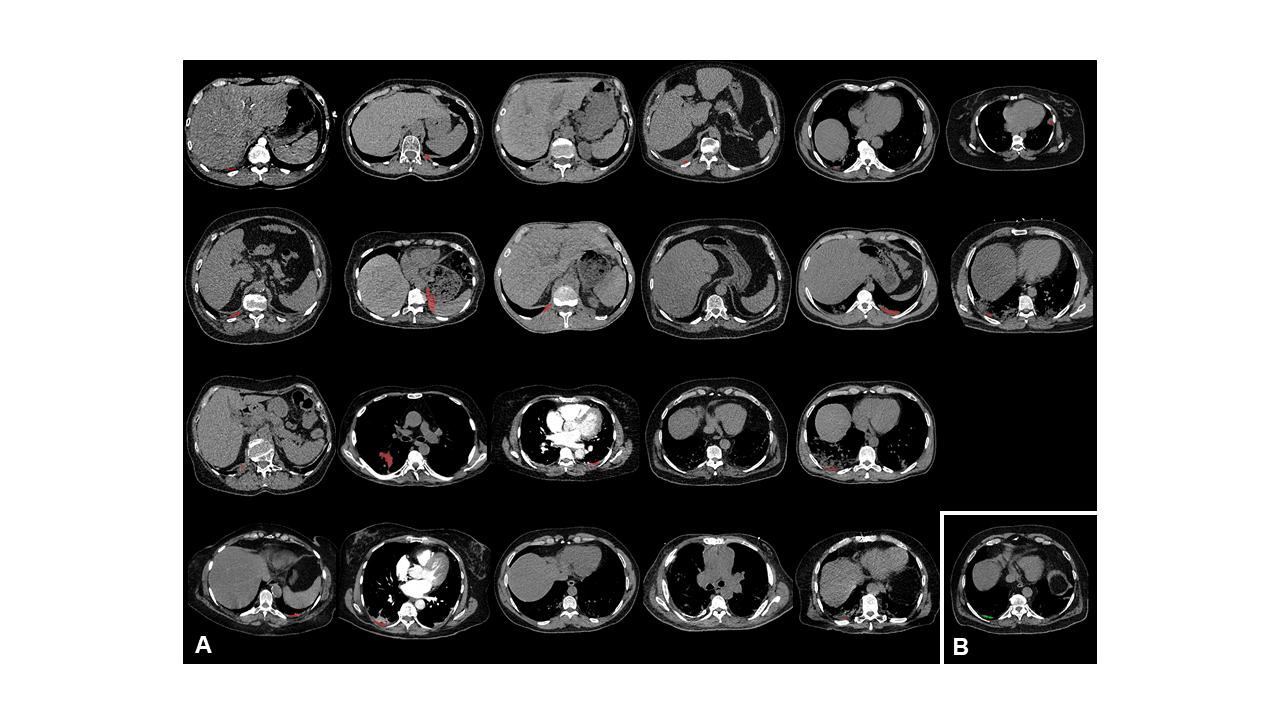

Supplement: Supplementary file 10 [file ir-57-552-s010.tif]

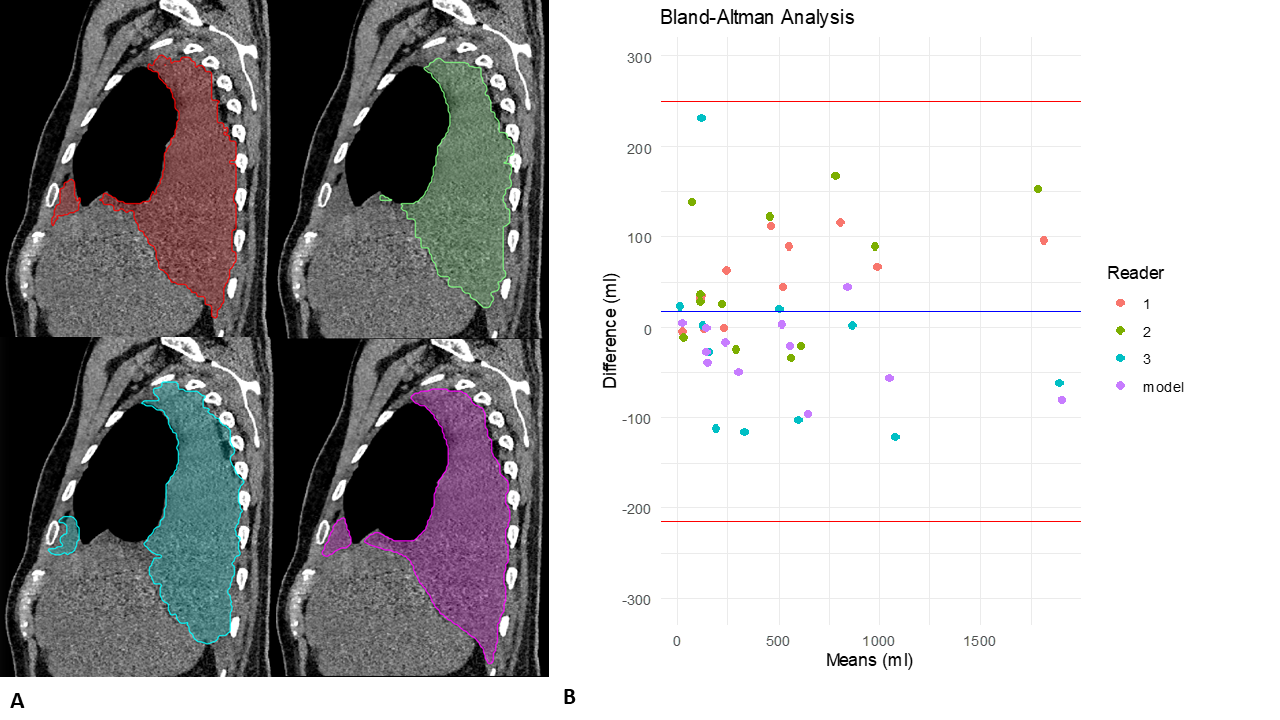

Supplement: Supplementary file 11 [file ir-57-552-s011.tif]

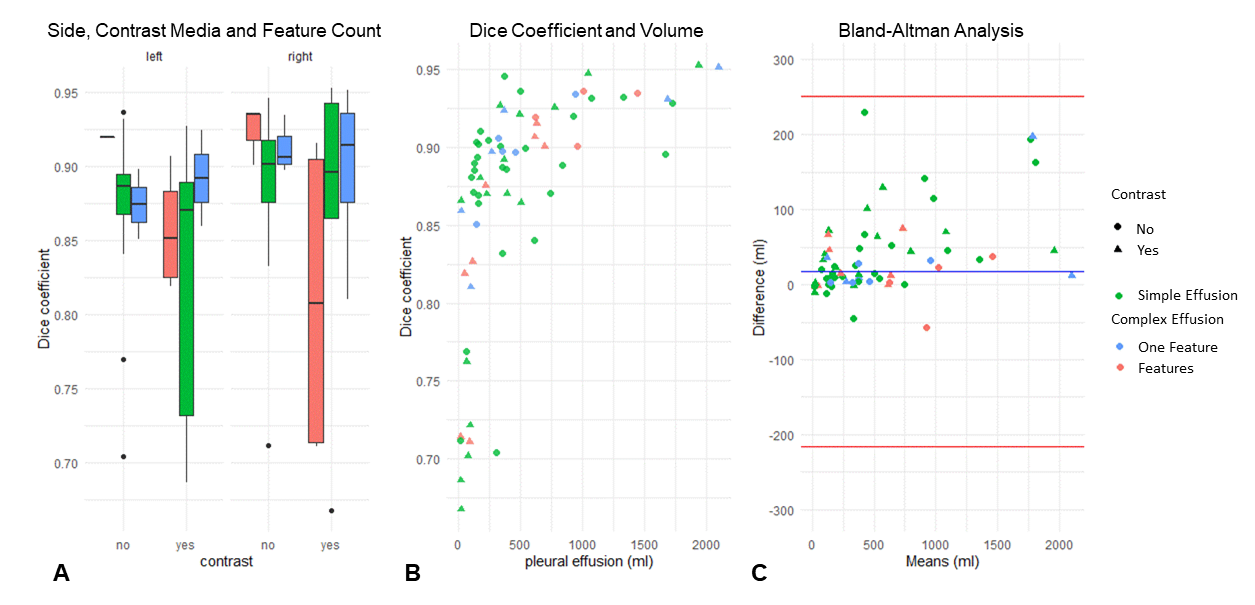

Supplement: Supplementary file 12 [file ir-57-552-s012.tif]

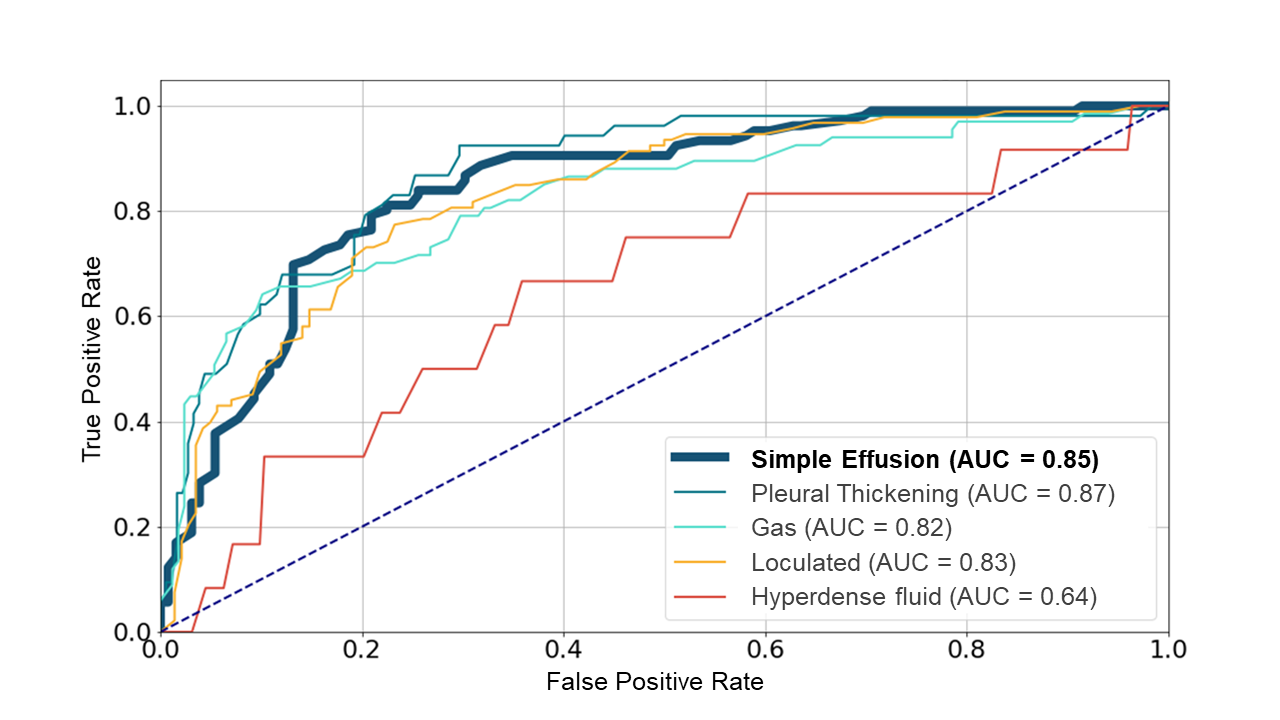

Supplement: Supplementary file 13 [file ir-57-552-s013.tif]
